# Utility of intermittent online quizzes as an early warning for residents at risk of failing the pediatric board certification examination

**DOI:** 10.1186/s12909-018-1366-0

**Published:** 2018-12-04

**Authors:** Rebecca Wallihan, Keely G. Smith, Mark D. Hormann, Rajesh R. Donthi, Kimberly Boland, John D. Mahan

**Affiliations:** 1 Nationwide Children’s Hospital, The Ohio State University School of Medicine, 700 Children’s Drive, Columbus, OH 43205 USA; 2 McGovern Medical School, The University of Texas Health Science Center, 6410 Fannin Street, Suite 500, Houston, TX 77030 USA; 3 McGovern Medical School, The University of Texas Health Science Center, 6431 Fannin Street, MSB 3.020, Houston, TX 77030 USA; 4 Children’s Hospital of Los Angeles and Keck School of Medicine of USC, 4650 Sunset Blvd Mailstop #94, Los Angeles, CA 90027 USA; 5 University of Louisville School of Medicine, 231 E Chestnut St, Louisville, KY 40202 USA

**Keywords:** Board preparation, Spaced learning, Test-enhanced learning, Testing effect

## Abstract

**Background:**

Traditionally, quizzes have been applied as a tool for summative assessment, though literature suggests their use as a formative assessment can improve motivation and content retention. With this premise, we implemented a series of intermittent, online quizzes known as the Board Examination Simulation Exercise (BESE). We sought to demonstrate an association between BESE participation and scores and performance on the American Board of Pediatrics (ABP) Certifying Examination (CE).

**Methods:**

Residents were assigned online quizzes on a single topic at 2 week intervals that consisted of 20 multiple choice questions written by the study authors. This analysis includes graduates of 3 Pediatric and Internal Medicine-Pediatrics residency programs.

**Results:**

Data were available for 329 residents. The overall BESE score weakly correlated with ABP CE score (*n* = 287; *r* = 0.39, *p* < 0.0001). ABP CE pass rates increased from 2009 to 2016 at all programs combined (*p* = 0.0001). A composite BESE score ≤ 11 had sensitivity of 54% and specificity of 80% for predicting ABP CE failure on the first attempt. There was no difference in ABP CE failure rates or scores by number of completed quizzes.

**Conclusion:**

Intermittent online quizzes implemented at three pediatric residency programs were associated with overall increasing ABP CE pass rates. BESE increased program emphasis on board preparation. Residents with lower BESE scores more often failed ABP CE. Though additional data are needed, BESE is a promising tool for pediatric resident learning and board preparation. It may also aid in earlier identification of residents at higher risk of failing the ABP CE and facilitate targeted interventions.

**Electronic supplementary material:**

The online version of this article (10.1186/s12909-018-1366-0) contains supplementary material, which is available to authorized users.

## Background

Traditional methods of teaching focus on the use of quizzes or tests as a summative assessment – assignment of a grade or score at the end of a learning activity. However, literature suggests the use of quizzes as a formative assessment, i.e. with the goal of monitoring progress and providing ongoing feedback, can improve motivation and content retention. Specifically, studies have demonstrated improved retention of material following repeated testing compared with either a single study period, repeated study without testing, or concept mapping [1–6]. The premise behind this is that in contrast to repeated study, testing requires active retrieval of information, a key component of long-term retention [2–4]. This is often referred to as the *testing effect* or *test-enhanced learning*. Additionally, recurring quizzes can take advantage of the spacing effect, in which spaced repetitions lead to better long-term retention than non-spaced repetitions [7–10].

Aside from content retention, formative assessment through quizzes can benefit both learners and teachers in a number of ways. Learners can identify areas of weakness and become accustomed to the exam timing and format. Moreover, quizzes can improve performance on summative assessments [2, 11, 12]. Teachers can assess the efficacy of curricula and instruction methods, as well as identify learners who may be struggling or at risk for failure, allowing early, targeted intervention [1].

In medical education, spaced and test-enhanced learning have been used to improve both content retention and skill performance for medical students and residents [5, 12–14]. Despite evidence of their effects on knowledge retention, little data exist on their value as a tool for preparation for board certification examinations. With the goal of helping pediatric residents pass their initial American Board of Pediatrics (ABP) Certifying Examination (CE), in 2011 we initiated a series of online quizzes known as the Board Examination Simulation Exercise (BESE). BESE was developed by two of the authors (JDM and KS), included multiple choice questions written and peer reviewed by the study authors, and quizzes utilized principles of both the spacing and testing effects. Five years after implementation, we sought to evaluate the utility of these quizzes as both a resident preparation tool for the ABP CE and a predictor of ABP CE performance. Specifically, we hypothesized that 1) higher resident participation in and performance on BESE quizzes would be associated with higher performance on the final year In-Training Examination (ITE) and the ABP CE, and 2) introduction of BESE would be associated with improved ABP CE board pass rates in the programs.

## Methods

### Settings and subjects

BESE was implemented in 2011 at Nationwide Children’s Hospital (NCH) and McGovern Medical School at The University of Texas Health Science Center (UTH), and in 2013 at University of Louisville (UL). Residents included in this initial analysis were Pediatrics or Internal Medicine-Pediatrics residents at NCH, UTH, and UL, completing residency between 2013 and 2016 for NCH and UTH and 2015–2016 at UL. Therefore, residents at each program had the opportunity to complete at least 2 years of quizzes. Residents who did not take the final year ITE were excluded from ITE analyses. Residents who did not take the ABP CE were excluded from the ABP CE analyses. This study was submitted to the NCH Institutional Review Board (NCH IRB #14–00720) and was determined to not meet the definition of human subjects research.

### Intervention

BESE structure has been previously described without outcome data [15]. Twenty-three online quizzes (22 in Year 1) were offered to all residents each academic year (July –June), approximately every 2 weeks. Each quiz consisted of 20 multiple-choice questions—drawn randomly for each participant from a test item bank of 40–50 questions per topic area—to be completed in 25 consecutive minutes. Question content was derived from ABP content specifications [https://www.abp.org/content/general-pediatrics-content-outline] and divided into 23 sections (Additional file 1: Table S1). Given the length of the ABP content specifications we did not aim to cover all of the content but utilized the quizzes to sample knowledge in a given topic area. Questions were written by JDM, KB, KGS, and MDH, and peer reviewed for accuracy by JDM, RRD, and RW. Question format was modelled after widely accepted guidelines (http://www.nbme.org/IWW/). Quizzes were taken online via each institution’s learning management system (LMS) providing a random selection of questions at each quiz. Feedback was provided immediately at the conclusion of each quiz. The score for each quiz was provided and the correct answer was revealed for each question, along with an explanation of the right and wrong answers with 2–5 paragraphs of teaching points. To minimize recall, the questions and answers were available to the participant for review for only 1 h after the quiz and were not able to be copied.

### Data collection and definitions

Individual resident BESE participation and scores were collected in each institution’s LMS. ITE and ABP CE performance were routinely made available to each Program Director from the ABP. Resident data collected included the number and topics of quizzes completed each year, raw score on each quiz, ITE scores, and first attempt ABP CE score. Data were deidentified by program coordinators at each site prior to aggregation and analysis by the authors.

BESE years were categorized according to academic year. Residents were categorized into one of the following groups: Post Graduate Year (PGY) 1, PGY-2, or PGY-3/4. Participation was defined as the number of quizzes completed per year. Quiz score was the number of correct responses, with a maximum score of 20. The annual BESE score was the mean of a resident’s quiz scores during a given academic year. The composite BESE score was the mean of a resident’s scores during all years of training. The PGY-1 BESE score was the mean of a resident’s scores during their first year of training. Quizzes not taken were not included in score calculations.

### Outcomes

The primary outcome was failure on first attempt of the ABP CE. Secondary outcomes were final ITE score and ABP CE score on first attempt. The final ITE score was the percentage of correct answers on the ITE in the final year of training. The ABP CE score was the scaled score reported by the ABP.

### Data analysis

Normally distributed continuous variables were compared using the *t*-test or one way Analysis of Variance (ANOVA) and results expressed as means and standard deviation (SD). Non-normally distributed continuous variables were compared using the Mann–Whitney or Kruskal-Wallis tests and results expressed as medians and 25–75% interquartile range (IQR). Correlations were performed using Spearman’s test. Correlation coefficients 0.3–0.5 were considered weak, 0.5–0.7 moderate, and > 0.7 strong. Contingency tables were analyzed using Chi-square or Fisher’s exact test, as appropriate. Receiver Operating Characteristic (ROC) curves were created to determine the diagnostic ability of BESE as a tool to predict failure of the ABP CE. Since the prevalence of failure may change over time, positive and negative predictive values were computed for hypothetical prevalence values. Statistical analyses were performed using GraphPad Prism version 7 (La Jolla, CA).

## Results

Data were available for 329 residents – all eligible residents across the three programs: 173 (53%) NCH, 106 (32%) UTH, and 50 (15%) UL. Final year ITE scores were unavailable for 5 (1.5%) residents and ABP CE scores were unavailable for 37 (11%) residents due to individual decisions to not take the ABP CE. As shown in Table 1, the median number of quizzes completed per year was 15 [IQR 7–20], with PGY-1 and -2 residents completing more quizzes compared with PGY-3/4 residents. Further data on participation and scores are shown in Table 1.

**Table 1:** Number of residents, quizzes completed, and annual scores during the study period

	Year					
	2011–12	2012–13	2013–14	2014–15	2015–16	All Years
Number of residents, n						
NCH	92	137	133	90	46	173
UTH	24	64	55	48	27	106
UL	-	-	50	50	25	50
All Residents	116	201	238	188	98	329
Number of quizzes completed, Median (IQR)^a^						
PGY-1	15 (11–17)	22 (19–22)	19 (7–21)	-	-	18 (12–22)
PGY-2	13 (9–15)	20 (16–22)	18 (7–21)	18 (13–21)	-	17 (10–21)
PGY-3/4	7 (4–9)	16 (9–20)	14 (8–19)	8 (2–15)	8 (2–12)	10 (3–17)
All Residents	13 (9–16)	20 (14–22)	17 (8–21)	12 (5–18)	8 (2–12)	15 (7–20)
Resident Annual Score, Median (IQR)^b^						
PGY-1	11.1 (10.3–11.8)	11.1 (10.3–11.8)	10.4 (7.8–11.3)	-	-	11.2 (10.3–11.9)
PGY-2	11.8 (11–12.7)	11.7 (11–12.5)	12 (11–12.9)	11.6 (11–12.6)	-	11.9 (11–12.7)
PGY-3/4	11.2 (10.6–12.2)	12.5 (11.4–13.1)	12.1 (11–13.6)	12.2 (11.1–13.3)	11.7 (10.7–12.7)	12 (11–13.1)
All Residents	11.5 (10.6–12.4)	11.7 (11–12.6)	11.9 (11–12.9)	12 (11.0–13.1)	11.7 (10.7–12.7)	11.8 (11–12.8)

*PGY* Post Graduate Year, *NCH* Nationwide Children’s Hospital, *UTH* McGovern Medical School at The University of Texas Health Science Center, *UL* University of Louisville

^a^Number of quizzes completed lower in PGY-3/4 group compared with either PGY-1 or − 2; Kruskal-Wallis with Dunn’s Multiple Test Correction, *p* < 0.0001

^b^PGY-1 scores lower than either PGY-2 or − 3/4 scores, and PGY-2 scores lower than PGY-3/4 scores; Kruskal-Wallis with Dunn’s Multiple Test Correction, *p* < 0.0001

**Fig. 1:**
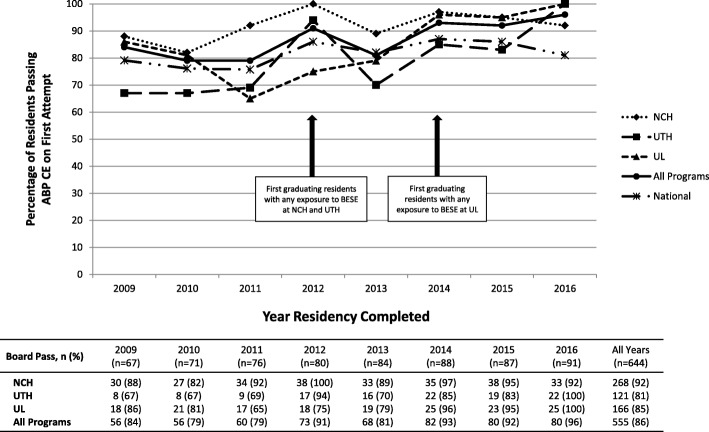
Percentage and number of residents passing the ABP CE on the first attempt. The graph above displays the percentage of residents passing the ABP CE on the first attempt for Pediatric and IM-Pediatric residents completing residency 2009–2016 from NCH (diamonds and dotted line), UTH (squares and long-dashed line), UL (triangles and short-dashed line), and all BESE programs (black circles and solid line). National first-attempt pass rates are represented by the asterisks and dashed-dotted line. The table beneath displays the number and percentage of residents passing the ABP CE on the first attempt. BESE quizzes were implemented in 2011 at NCH and UTH and in 2013 at UL. There were significant increases in the pass rate from 2009 to 2016 at UTH (p = 0.009), UL (p = 0.003), and all programs combined (p = 0.0001), χ^2^ for trend. NCH=Nationwide Children’s Hospital; UTH = McGovern Medical School at The University of Texas Health Science Center; UL = University of Louisville; ABP CE = American Board of Pediatrics Certifying Examination

Overall, 26 (9%) residents failed the ABP CE on the first attempt. There were no differences in ABP CE failure rates or scores by number of quizzes completed, either in the final year or throughout training (data not shown). ABP pass rates significantly increased from 2009 to 2016 at UTH (*p* = 0.009), UL (*p* = 0.003), and all programs combined (*p* = 0.0001), χ^2^ for trend. Further data on ABP CE pass rates are shown in Fig. 1.

**Fig. 2:**
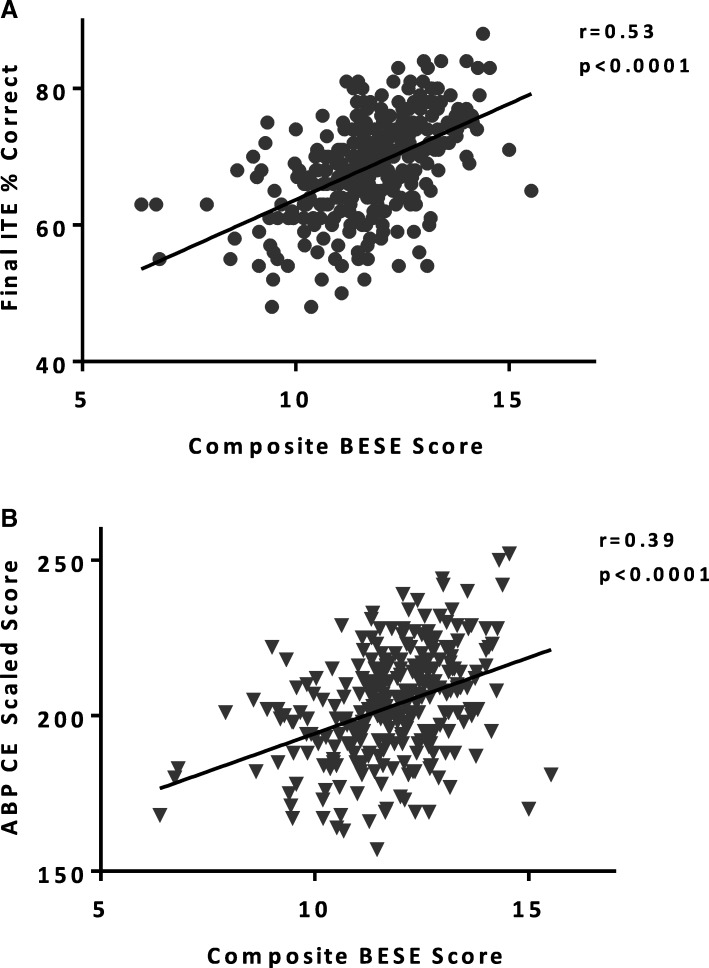
Correlation of Composite BESE Scores with ITE and ABP CE Scores. The composite BESE score showed weak to moderate correlation with both final year ITE (**a** – circles, n = 318) and 1st attempt ABP CE scores (**b** – inverted triangles, n = 287); Spearman correlation. ITE = In-Training Examination; ABP CE = American Board of Pediatrics Certifying Examination

As shown in Fig. 2, there was an association between the composite BESE score and both the final ITE score (*n* = 318, *r* = 0.53, *p* < 0.0001) and ABP CE score (*n* = 287, *r* = 0.39, p < 0.0001). The PGY-1 BESE score was also associated with the final ITE score (*n* = 190, *r* = 0.47, *p* < 0.0001) and the ABP CE score (*n* = 181, *r* = 0.44, *p* < 0.0001). Rates of ABP CE failure increased with composite score quartile rank, with 3, 5, 7, and 19%, of residents failing the ABP CE in quartiles 1 (top) - 4 (bottom), respectively (*p* = 0.0005, χ^2^ for trend). Residents with composite scores in the bottom quartile were more likely to fail the ABP CE than residents in the top 3 quartiles combined [OR 4.6 (95% CI: 1.9–10.3), *p* = 0.006].

The PGY-1 and composite BESE scores performed similarly in predicting failure of the ABP CE. The area under the curve (AUC) for PGY-1 Score was 0.77 (*p* = 0.0011), indicating fair accuracy in predicting ABP CE failure. The AUC for Composite Score was 0.69 (*p* = 0.013), indicating poor accuracy. A cutoff score of 11 was chosen as this is nearest to the 25th percentile of all composite scores (11.02). Table 2 shows the sensitivity and specificity of PGY-1 and composite scores ≤11 for failure of the ABP CE, as well as positive and negative predictive values at hypothetical prevalence values.

**Table 2:** Positive and Negative Predictive Values for BESE Scores at Hypothetical Prevalences of ABP CE Failure

Cutoff Value (Raw Score)	Sensitivity (%)	Specificity (%)	Hypothetical prevalence of failure (%)	Positive Predictive Value (%)	Negative Predictive Value (%)
PGY-1 BESE Score ≤11	67	62	5	8	97
			10	16	94
			15	24	91
			20	31	88
Composite BESE Score ≤11	54	80	5	12	97
			10	23	94
			15	32	91
			20	40	87

*ABP CE* American Board of Pediatrics Certifying Examination

## Discussion

Our programs incorporated online quizzes into the educational curriculum of our pediatric and internal medicine-pediatrics residents with the goal of improving performance on the ABP CE. Participation was not associated with performance on the ABP CE. Composite BESE scores were associated with both the final year ITE and ABP CE scores. Furthermore, composite scores in the bottom quartile were associated with a higher risk of failure on the ABP CE. Most importantly, overall board pass rates improved for the 3 programs combined and specifically for UTH and UL during the period of BESE implementation. While national pass rates for first time test-takers also increased during this time, all programs were above national rates in 2016.

Studies show that both medical knowledge and clinical skills decay with time. Medical students retain approximately 40% of basic science while still in medical school and approximately 25% after 50 years of practice [16]. In a study of cardiopulmonary resuscitation (CPR) skills, only 2.4% of those trained 3 years earlier could successfully perform CPR [17]. However, spaced and test-enhanced learning can diminish knowledge loss and enhance medical education at all levels. Among medical students, the use of spaced education on urology topics was associated with higher scores on an end-of-year test [18, 19]. In another study, students performed better on a resuscitation skills assessment if they were tested at the end of training compared with those who were not tested, [14] and a difference was preserved 6 months following the intervention [20]. Residents and faculty have shown both short [5, 21–23] and long term [24] improvements in medical knowledge following spaced or test-enhanced education. Beyond knowledge retention, these principles can change practice. Kerfoot et al. used spaced learning to decrease the percentage of inappropriate prostate specific antigen screening [25]. Matzie and colleagues demonstrated improvements in the frequency and quality of feedback to medical students when surgical residents received spaced emails containing teaching points on feedback [26].

While multiple studies have demonstrated the benefits of spaced, test-enhanced learning in medical education, little data exist on its association with outcomes on board certification examinations. Peterson et al. examined the effects of self-assessment modules on certification exam results among family medicine residents. Module completion during residency was associated with both increased odds of passing the certification exam and a higher score [27]. However, the authors only evaluated completion of the modules and did not correlate module scores with exam outcomes. In the present study, we identified a cutoff for both PGY-1 and composite scores associated with increased odds of failing the ABP CE. The first year risk stratification is particularly important for earlier identification and intervention for residents at risk. ITEs may serve a similar purpose but are only offered annually and specific question data are not reported. The frequency of BESE provides learners continual assessment with immediate, more specific feedback.

There are many strengths to the curriculum structure described. First, the quizzes mimic the ABP CE format and time-per-question. This is important, as formative assessments are most effective when the format matches the summative assessment [1]. Second, rapid scoring provides immediate, explanatory performance feedback to residents [2]. Third, quiz organization by topic and repetition annually allows residents to track longitudinal performance in specific subjects. Both residents and program directors can use these results to identify areas of weakness for targeted intervention. Normative data allows peer performance comparison. Finally, the use of spacing and testing effects promotes long term retention.

There are also some limitations to this study. First, ITE and ABP CE scores in these learners are influenced by multiple factors, including different program educational activities and individual board preparation routines. Second, BESE participation and scores may reflect general attitudes toward board preparation. Residents who took BESE seriously may have prepared for the certification exam more intensely than those who did not. Nevertheless, identifying these residents early in training who display lower performance can still be beneficial to program directors. Third, we recognize that standardized test scores may better reflect test-taking abilities and not capture true medical knowledge. Furthermore, we know that these results may not indicate differences in clinical skills or effectiveness. Additionally, since BESE was designed to sample resident knowledge in specific topic areas we cannot make a statement on their comprehensive knowledge in any area. Fourth, only two of the three programs saw statistically significant increases in ABP CE pass rates during the study period. This may have been due to the ceiling effect, as pass rates at NCH were already above national rates prior to BESE implementation and were the highest among the three programs. Finally, test validity has not yet been established and the small number of items may affect the reliability and validity of the quizzes. Though the questions were peer-reviewed for accuracy prior to use, item quality may have been variable. Future plans include performing test item analysis and validation of these questions, and extending this process to additional residency programs to better define its generalizable impact.

## Conclusion

Using five years of data from three residency programs, we demonstrated an association between performance on biweekly quizzes and pediatric board certification exam results and, more importantly, improved board pass rates over time. Though additional data are needed, BESE is a promising approach for resident learning and board preparation. Residency programs should consider incorporation of spaced, test-enhanced learning sessions into their curricula, simulating high stakes examination conditions for their discipline.

## Additional file

Additional file 1: **Table S1.** Content distribution for BESE quiz topics. Distribution of American Board of Pediatrics (ABP) content areas across 23 BESE quizzes. The first (left) column displays the individual quiz number; the second column displays the ABP content areas from which questions for each quiz were derived; the final column displays the approximate percentage of the ABP Certifying Examination (CE) content from each area. (DOCX 15 kb)

## Abbreviations

ABP: American Board of Pediatrics
ANOVA: Analysis of Variance
AUC: Area Under the Curve
BESE: Board Examination Simulation Exercise
CE: Certifying Examination
CPR: cardiopulmonary resuscitation
IQR: Interquartile Range
ITE: In-Training Examination
LMS: learning management system
NCH: Nationwide Children’s Hospital
PGY: Post Graduate Year
ROC: Receiver Operating Characteristics
UL: University of Louisville
UTH: McGovern Medical School at The University of Texas Health Science Center
