# Supplementary material for: Utility of intermittent online quizzes as an early warning for residents at risk of failing the pediatric board certification examination
Source: BMC Med Educ. 2018 Dec 4;18:287. doi: 10.1186/s12909-018-1366-0 (PMC6278081; doi:10.1186/s12909-018-1366-0)
Supplement: Supplementary file 1 — Table S1. Content distribution for BESE quiz topics. Distribution of American Board of Pediatrics (ABP) content areas across 23 BESE quizzes. The first (left) column displays the individual quiz number; the second column displays the ABP content areas from which questions for each quiz were derived; the final column displays the approximate percentage of the ABP Certifying Examination (CE) content from each area. (DOCX 15 kb) [file 12909_2018_1366_MOESM1_ESM.docx]

Table S1. Content distribution for BESE quiz topics

| Quiz | ABP Content Specifications | % of ABP CE |
| --- | --- | --- |
| 1 | Growth and Development | 5% |
| 2 | Nutrition and Nutritional Disorders | 4% |
| 3 | Preventive Pediatrics | 5% |
| 4 | Poisoning and Environmental Exposure to Hazardous Substances and Pharmacology | 4% |
| 5 | Fetus and Newborn Infant and Fluid and Electrolyte Metabolism | 6% |
| 6 | Genetics and Dysmorphology and Metabolic Disorders | 4% |
| 7 | Allergy and Immunologic Disorders and Disorders of Blood and Neoplastic Disorders | 6% |
| 8 | Infectious Diseases | 4.5% |
| 9 | Endocrine Disorders | 3.5% |
| 10 | Gastrointestinal Disorders | 3.5% |
| 11 | Respiratory Disorders | 4% |
| 12 | Cardiovascular Disorders | 3% |
| 13 | Renal Disorders and Genital System Disorders | 4% |
| 14 | Neurologic Disorders | 3% |
| 15 | Musculoskeletal Disorders | 3% |
| 16 | Skin Disorders and Collagen Vascular & Other Multisystem Disorders | 5% |
| 17 | Disorders of the Eye, Ear, Nose, and Throat | 5% |
| 18 | Adolescent Medicine/Gynecology and Sports Medicine and Physical Fitness | 6.5% |
| 19 | Substance Abuse and Psychosocial Issues and Problems | 4.5% |
| 20 | Critical Care and Emergency Care | 5% |
| 21 | Disorders of Cognition, Language, Learning, and Attention | 3.5% |
| 22 | Behavioral and Mental Health Issues | 4% |
| 23 | Statistics, Ethics, and Patient Safety and Quality Improvement | 4% |

ABP=American Board of Pediatrics; CE=Certifying Examination
